# HBO1 as an Important Target for the Treatment of CCL4-Induced Liver Fibrosis and Aged-Related Liver Aging and Fibrosis

**DOI:** 10.1155/2022/1881519

**Published:** 2022-12-06

**Authors:** Baopeng Xing, Hainan Lan, Haifeng Li

**Affiliations:** ^1^Emergency Medicine Department, First Hospital of Jilin University, Changchun 130031, China; ^2^College of Animal Science and Technology, Jilin Agricultural University, China

## Abstract

The liver is the largest digestive organ in the human body. The increasing incidence of chronic liver fibrosis is one of the major health challenges in the world. Liver fibrosis is a wound-healing response to acute or chronic cellular damage of liver tissue. At present, despite a series of research progress on the pathophysiological mechanism of fibrosis that has been made, there is still a gap in identifying antifibrotic targets and converting them into effective treatments. Therefore, it is extremely important to seek a molecular target that can alleviate or reverse liver fibrosis, which has important scientific and clinical significance. In the current study, to evaluate the therapeutic effect of HBO1 as a molecular target on liver aging and fibrosis, naturally-aged mice and CCL4-induced liver fibrosis mice were used as animal models, and multiple experiments were performed. Experimental results showed that HBO1 knockdown could strongly mitigate the accumulation of hepatic collagen by Masson and Sirius Red staining. Further study showed that HBO1 knockdown reduced the expression of fibrosis-related marker molecules (*α*-SMA, collagen type I (ColI), and fibronectin). Further work showed that HBO1 knockdown could significantly alleviate HSC activation. On this basis, we analyzed the underlying mechanism by which HBO1 alleviates liver fibrosis. It was found that HBO1 knockdown may modulate liver fibrosis by regulating the processes of EMT, inflammation, and oxidative stress. We further studied the effect of HBO1 knockdown on liver aging and aging-related liver fibrosis, and the results showed that HBO1 knockdown could significantly reduce the level of aging-related liver fibrosis and relieve liver aging. In conclusion, we systematically investigated the potential of HBO1 as a therapeutic target to attenuate liver fibrosis and liver aging. The current study found a crucial target for liver fibrosis and liver-aging therapy, which has laid a solid foundation for the liver fibrosis-related research.

## 1. Introduction

The liver is a solid digestive organ, occupying most of the right upper quadrant and part of the left upper quadrant in the body cavity, accounting for about 2% of body weight [1]. There are about a thousand chemical reactions in the body that take place in the liver; therefore, the liver is also known as the “processing factory” of the human body and plays a very important role in life activities [1]. Most of the toxic substances are processed by the liver to become nontoxic or low-toxic; various nutrients such as protein, fat, carbohydrates, vitamins, and minerals in the daily intake of food are initially digested and absorbed in the gastrointestinal tract and then sent to the liver; after being decomposed, the substances and energy required for life activities in the body are synthesized again. Therefore, the liver is a very important organ for maintaining the health of the body. Liver fibrosis is a reversible wound-healing response to acute or chronic cellular damage to liver tissue, which reflects the balance between liver repair and scarring. At present, the incidence of chronic fibrotic liver disease is increasing, and about 2 million people die from it every year worldwide. The pathological repair response of the liver to chronic injury promotes the diffuse excessive deposition and abnormal distribution of hepatic extracellular matrix (collagen, glycoprotein and proteoglycan, etc.) lead to liver fibrosis. The etiology of liver fibrosis includes viral hepatitis and drug and alcoholic hepatitis. It is the result of a comprehensive pathological response of injury-inflammation-regeneration-repair, and it has become an inevitable stage for all chronic liver diseases to develop to cirrhosis [2]. Despite substantial progress in the pathophysiology of liver fibrosis, there is still a gap in identifying antifibrotic targets and translating them into effective treatments [3]. A range of potential molecular therapies targets has been discovered to treat liver fibrosis. The current main strategy is to target key molecules of liver fibrosis, such as type I procollagen or other key components of the ECM [4]. Furthermore, there are also studies targeting growth factor/chemokine receptors to treat liver fibrosis [5]. In addition, some plant extracts or bioactive molecules have also been found to have antifibrotic potential [6, 7]. Recently, some studies have shown that noncoding RNAs also have antifibrotic effects [8]. But so far, there is no effective antifibrotic therapy that can successfully reverse the process of liver fibrosis.

Aging is also closely related to liver fibrosis, and aging is also one of the pathogenesis of liver fibrosis. Therefore, the search for molecular targets that can reduce or reverse liver fibrosis has extremely important potential clinical significance.

Histone acetyltransferase (HAT) is an important protease for acetylation, which can transfer the acetyl group of acetyl-CoA to a specific lysine residue at the N-terminus of histones, also known as HBO1 (KAT7/MYS2); its cDNA sequence encodes a 611 amino acid protein with a molecular weight of about 83 kDa [9, 10]. Acetyltransferase not only binds to transcription factors to enhance or inhibit its transcriptional activity but also acts as a coactivator and participates in the regulation of gene replication initiation. As a cofactor of transcription factors, HBO1 can enhance the transcriptional activity of steroid hormone receptors. Acetylation and deacetylation of histones is a dynamic process closely related to gene activation and repression. Hyperacetylation means transcriptional activity, while hypoacetylation is associated with transcriptional repression. HBO1 has important biological activities. For example, it has been reported that HBO1 is involved in complex biological functions in cells, including gene transcription, DNA damage repair, and apoptosis [11]. Furthermore, it has been reported that HBO1 may be involved in cancer initiation and progression [12]. Despite substantial progress on the pathophysiological mechanisms of liver fibrosis, there is still a gap in identifying antifibrotic targets and translating them into effective treatments.

In this work, we used two liver fibrosis models. One is a model of natural aging-related liver fibrosis, and another is a model of CCL4-induced liver fibrosis. We systematically evaluated the potential of HBO1 as a target to treat liver fibrosis, liver aging, and aging-related liver fibrosis. It was found that the knockdown of HBO1 can remarkably mitigate liver fibrosis and liver aging, and the current study shows that HBO1 is an important target for liver fibrosis and liver-aging therapy.

## 2. Materials and Methods

CCL4 was purchased from Sigma (#488488). Olive oil (#PHR2902) from Merck. BCA protein quantitative kit Sirius Red reagent (#365548) and Masson (#1.00485) were purchased from Merck Biotechnology Co., Ltd. Xylene and alcohol were purchased from Huacheng Biology Co., Ltd. BSA powder was purchased from Solarbio Co., Ltd. Dulbecco's modified basic medium (DMEM, #11965118) was from Gibco (USA). *α*-SMA (Cat No. 14395-1-AP, 1 : 1000 dilution), TNF *α* (Cat No. 60291, 1 : 1000 dilution), TGF-*β*1 (Cat No: 21898-1-AP, 1 : 1000 dilution), and anti-fibronectin (Cat No. 66042-1-Ig, 1 : 500 dilution) were purchased from Proteintech Company (Wuhan, China). Anticollagen (ab270993, 1 : 800 dilution) was from Abcam (Cambridge, UK).CoraLite488-conjugated goat anti-rabbit IgG (H+L) was from Proteintech Company (Wuhan, China). Mouse IL-1*β* ELISA kit (#PI301), mouse TNF-*α* ELISA kit (#PT512), and mouse IL-6 ELISA kit (#PI326) were purchased from Beyotime Co., Ltd. Western blot and IP cell lysate were purchased from Huacheng Co., Ltd. Protein-loading buffer and protein prestaining marker were purchased from Thermo Fisher Technology (USA).

### 2.1. CCL4-Induced Liver Fibrosis Model

The experimental animal protocol was approved by the Animal Ethics Committee of the Jilin Agricultural University. Male C57 mice weighing 18-22 g (8-week-aged males) were randomly divided into experimental groups (*n* = 10) and the control group (*n* = 10); preparation of CCL4 solution, the volume of CCL4: olive oil was prepared according to the ratio of 1 : 4, the mice from the model group were injected intraperitoneally with the mixed solution at 0.6 *μ*l/g twice per week for 6-8 weeks.

### 2.2. Sirius Red Staining

The tissue sections were placed in a 60°C oven for 40 min. The tissue sections were then placed in the xylene solution for 10-15 min. Sections were dewaxed with gradient alcohol to water, washed with distilled water for 2 min, dripped with Sirius Red staining solution, and placed in a wet box, stained at room temperature for 60 min, and then rinsed off the surface of the tissue with running water. After dehydration in absolute ethanol for 30 s, the samples were transparent with xylene, and the sliced samples were mounted in neutral gum. The sections were observed and photographed under a microscope.

### 2.3. Indirect Immunofluorescence

The tissue sections were washed with TBS for two times (5 min each time). Tissues were blocked with blocking solution (10% serum+1% BSA+0.3% triton-100) and incubated for 2 h at room temperature. Antibodies were incubated with tissue sections at 4°C for 12 h. After washing, the secondary antibody was then added and kept at 4°C for 12 h. Samples were analyzed using CLSM (FV3000).

### 2.4. Cell Proliferation Detection via MTT

Cell densities were seeded in 96-well cell culture plates (1 × 10^4^ per well). The cells were cultured for 10 h and then treated with CCL4 or LPS (6 replicate wells for each concentration), and the remaining wells were filled with PBS as a blank control. The 96-well culture plates were then placed in a CO_2_ incubator for 24 h. After 24 hours, MTT solution was prepared with DMEM at a concentration of 5 mg/ml. 20 *μ*l of MTT solution was then added to each well, and the culture was continued for 3 h. After 3 h, the original medium was discarded, 150 *μ*l of DMSO solution was added to each well, placed on a multifunctional shaker, and shaken at low speed for 10 min to completely dissolve the formazan crystals. Absorbance was measured using an ELISA reader (OD-450 nm).

### 2.5. Cell Culture

Cells were cultured using DMEM supplemented with 10% serum. AML12 (CRL-2254) was purchased from ATCC. The cells were recovered from the liquid nitrogen tank and then cultured in a suitable incubator (5% CO2, 37°C). The cells used in the experiment were all in logarithmic growth phase with good growth conditions.

### 2.6. Cell Cycle Analysis by Flow Cytometry

Cells were collected by centrifugation for 10 min (1000 rpm). After the cell samples were centrifuged, the supernatant was discarded. The cell pellet was homogeneously pipetted, and the cell samples were fixed with fixative solution (90% ethanol: 10% PBS: 1% serum) at 4°C overnight. After the cells were fixed, the cells were centrifuged at low speed (1000 rpm) for 10 min and discarded. The supernatant was then washed twice with PBS containing 3% calf serum; the supernatant was discarded, 0.2 ml of RNase A (1 mg/ml) enzyme was added and incubated at 37°C for 30 min, then PI was added for 20 min in the dark. Cell cycle was detected by flow cytometry.

### 2.7. Apoptosis Assay

The cells were washed twice with PBS, and then digested with 0.05% trypsin. The cells were placed at room temperature for about 2 min. After the cells were digested, the digestion was terminated with a medium containing FBS. The cell suspension was then centrifuged. The cells were then washed once with precooled PBS, and the cell samples were centrifuged (300 g for 3 min). The cell pellet was then collected. Annexin V-FITC/Propidium Iodide (PI) solution was added according to manufacturer's instructions. The cells were then resuspended and incubated for 15 min at RT. After washing, cell samples were determined using a flow cytometer (BD).

### 2.8. Immunofluorescence Staining for Cell

The cells were seeded in a 15 mm petri dish at a density of 5 × 10^4^/well, and the cells were distributed at the bottom of the petri dish. After adherence, cells were cultured for 24 h. The cell culture medium was discarded. The cells were then fixed and permeabilized. 50 *μ*l of 5% goat serum was added to each well of the cell culture plate for 30 min; 50 *μ*l/well of primary antibodies such as *α*-SMA, collagen-I, NLRP3, and IL-1*β* were added and incubated overnight at 4°C in a wet box. The next day, the culture dish was taken out and the primary antibody was recovered. After washing 3 times with PBS, the corresponding fluorescently labeled secondary antibody was added and incubated for 120 min. Finally, nuclei were stained with DAPI. Cell information was collected under an inverted microscope and confocal microscope.

### 2.9. Western Blotting

After trypsinizing the adherent cells in the culture dish, the cell samples were centrifuged at 4°C for 5 min (1500 rpm); the supernatant was discarded. The centrifuged cells were added to RIPA lysis buffer and placed 30 min on ice, then SDS was added and boiled in boiling water for 10-15 min. The protein samples were then subjected to SDS-PAGE and transferred to PVDF membrane. Membranes were blocked by skim milk (5%) at 4°C for 12 h. Diluted primary antibodies were added and incubated at 4°C for 12 h. After primary antibody incubation, the membrane was washed for 3 times. After washing, BSA-diluted secondary antibody was added and incubated for 2 h at 37°C. The membrane was washed three times with TBST, and then the immunoprotein bands were exposed using Bio-Rad fluorescence system.

### 2.10. Coimmunoprecipitation (IP)

Cells were collected by centrifugation at 3000 rpm for 5 min. Then, 0.5 ml of the prepared IP buffer was added and lysed on ice for 30 min; after washing the beads with IP buffer, the samples were centrifuged at 3000 rpm for 5 min at 4°C. The lysed cells were then sonicated three times at 4°C. After centrifugation at 12000 rpm for 10 min, the supernatant was collected and mixed with the washed beads, and incubated at 4°C for 4-6 h. After the supernatant was discarded, an equal volume of SDS with beads was added as an IP sample for subsequent western blot analysis.

### 2.11. RT-PCR

TRIzol solution (1 ml) was added into the cells or tissues, the samples were mixed thoroughly at room temperature for 5 min, after which 0.2 ml of chloroform was then added at room temperature for 3-5 min. RNA pellets were collected and reverse-transcribed into cDNA. Then, RT-PCR assays were done using the primers shown in Supplementary Table 1.

### 2.12. Mitochondrial Membrane Potential Detection

The decrease of mitochondrial membrane potential occurs in the early stage of cell apoptosis. JC-1 is used as a fluorescent probe to detect the mitochondrial membrane potential. We can judge the changes of mitochondrial membrane potential according to the different fluorescence colors emitted by JC-1. When JC-1 forms a polymer, it usually exists in the mitochondrial matrix. At this time, JC-1 can excite fluorescence, which indicates that the mitochondrial membrane potential is high. When mitochondrial membrane potential decreases, JC-1 exists in the form of monomer and can excite green fluorescence. We therefore detected changes in mitochondrial membrane potential by the transition of the JC-1 probe between red and green fluorescence.

### 2.13. Detection of Intracellular ROS Levels

Cells in the logarithmic growth phase were collected and adjusted to 1 × 10^6^/ml; DCFH-DA was added in a final concentration of 10 *μ*m for 0.5 h at 37°C with 5% CO_2_ (mixing the well every 3-5 min). The cells were washed three times with serum-free medium or PBS to remove DCFH-DA that did not enter the cells. The cells were collected, and then the cell samples were subjected to a flow cytometer to detect ROS (488 nm excitation wavelength, 525 nm emission wavelength).

### 2.14. Isolation of Mice Primary Hepatocytes

C57 mice were intraperitoneally injected with anesthetic. After anesthesia, the mice were fixed on the operating table, and the mice were dissected and exposed with sterilized surgical instruments. The portal vein were exposed; liver perfusion enters from the portal vein and exits from the inferior vena. Liver perfusion started: (1) 20-40 ml of PBS and (2) 20 ml of DMEM prewarmed at 37°C with 0.05% collagenase IV to fully digest the liver cells. After the perfusion, the liver was removed into a 10 cm culture dish. Repeatedly pipetting with a pipette to disperse the hepatocytes, filtering out tissue debris with a cell sieve, the cells were centrifuged at 800 g for 5 min. The cell samples were resuspended and washed with PBS twice; the medium was added and placed in a 37°C cell incubator to continue to culture until cells adhesion to the wall. The whole process operation needs to be fast to reduce the possibility of contamination.

### 2.15. Immunohistochemistry

The sections were then incubated with 10% normal goat serum and incubated for 1 h for blocking. Primary antibodies were added and incubated overnight at 4°C. Tissue samples were incubated with secondary antibodies. Then, diaminobenzidine (DAB) color development was carried out; the degree of color development was controlled under optical microscope; and the samples were dehydrated with gradient ethanol, transparent in xylene, and sealed with neutral gum. Microscopic observation was then performed.

### 2.16. Masson Staining

Sections were routinely dewaxed and stained with iron hematoxylin staining solution for 5-10 min. The samples were then differentiated with an acidic ethanol differentiation solution for 5-15 s, and then washed with running water. Sections were stained using Masson's staining solution for 3-5 min. After being washed with distilled water for 1 min, the samples were stained with ponceau-fuchsin staining solution for 5-10 min. After washing with phosphomolybdic acid solution for 1-2 min, the sample was washed with weak acid working solution for 1 min. The samples were directly placed in the aniline blue staining solution for 1-2 min. After washing with the prepared weak acid working solution for 1 min, the samples were dehydrated using 95% ethanol for three times (5-10 s each time). The slice is transparent with xylene for 3 times (1-2 min each time); the samples are mounted with neutral gum and observed under a microscope.

### 2.17. Open Field Test (OFT)

OFT experiments were performed in a quiet environment. Animals were placed in the bottom of the box while videography and timing were performed. After finishing experiments, the camera was stopped. The observation time can be set according to the experiment. The inner wall and bottom surface of the box were cleaned to avoid the residual information of the last animal (such as urine and smell) from affecting the next test result. Animals were then replaced to continue the experiment.

### 2.18. Elevated Plus Maze

At the start of the experiment, mice were placed in a maze and their activity were recorded for 5 min. A series of behavioral parameters were then observed and recorded. After the experiment, the mice were taken out, the arms were cleaned, and alcohol was sprayed to remove the odor. Finally, data analysis was performed with ANY-maze behavioral software. The number of times of entering the open arm and the dwell time were negatively correlated with the mice's anxiety.

### 2.19. Y-Maze

The mice were placed in the starting arm and moved freely in the three arms for 5 min; the time and shuttle times of each mouse in each arm within 5 min were recorded by camera system. Data statistics: in the Y-maze experiment, the time that the mouse stayed between each arm within 5 min.

### 2.20. Construction of Lentiviral Vector and Lentivirus Injection

The plasmid lentiCRISPR v2 was purchased from Addgene (USA). The targeting sgRNA sequences (for HBO1) or sgNTC (control) were cloned into the lentiCRISPR v2. The lentiviral vector, psPAX2, and pMD2.G were cotransfected into HEK293 cells. After transfection, the virus particles were collected by centrifugation at 125000 g for 2 h at 4°C. sgRNA viruses were analyzed by counting the cell clones. The mice were injected with 200 *μ*l of lentivirus via tail vein (1 × 10^8^ PFU/ml).

### 2.21. Statistical Analysis

Data are expressed as the mean ± standard deviation (SD). Two-group comparisons were assessed using Student's *t*-test; comparisons of the means of 3 or more groups were analyzed by ANOVA. A value of p < 0.05 was considered to indicate a statistically significant difference.

## 3. Results

### 3.1. CCL4-Induced Liver Fibrosis Was Attenuated in the Presence of HBO1 Knockdown

**Figure 1:**
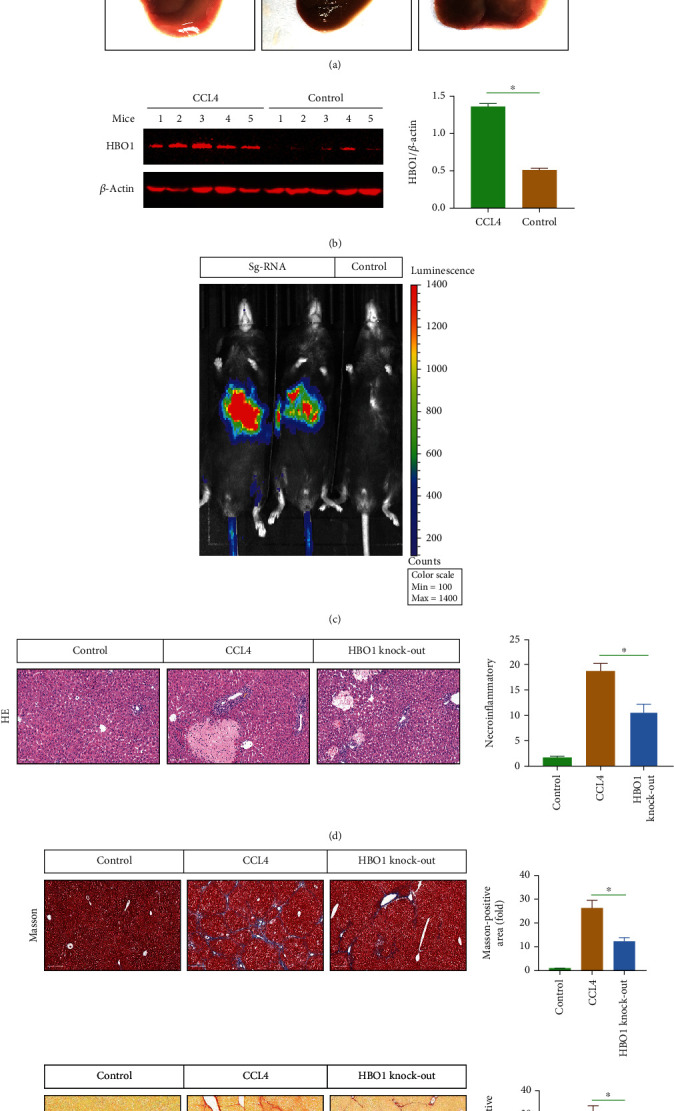
(a) HBO1 knockdown alleviated the pathological changes of the liver. (b) HBO1 expression was elevated by western blot in the CCL4-induced liver model. The protein samples were then subjected to SDS-PAGE and transferred to PVDF membranes. The immunoprotein bands were exposed using Bio-Rad fluorescence system. (c) Lentivirus was mainly enriched on liver tissue after injection. (d) HBO1 knockdown group could reduce liver damage in the mice by HE staining. (e) HBO1 knockdown group could reduce collagen deposition compared to the control group. Data are showed as mean ± standard deviation (SD). Asterisks indicate significant differences (^∗^*p* < 0.05).

Intraperitoneal injection of CCL4 can induce liver injury and fibrosis. For this, we selected C57 mice and randomly divided into two groups. The experimental group was injected with CCL4, and the control group was given with olive oil. The mice were injected with CCL4 twice a week; the liver tissue was taken for observation 7-8 weeks later. The results of immunohistochemical staining showed that the mice in the experimental group injected with CCL4 exhibited obvious liver fibrosis.  Figure 1(a) shows that, in the control group, after direct exposure of the liver, the surface of the liver in the control group was smoother and more uniform in color. In the liver fibrosis model group, the liver was obviously granular and sticky with surrounding tissues. The appearance of HBO1 knockout (sg-HBO1) was between the above two groups. Furthermore, the HBO1 expression was elevated in the CCL4-induced liver model (Figure 1(b)). In addition, lentivirus was mainly enriched on liver tissue after virus injection (Figure 1(c)); HBO1 expression was evaluated after lentivirus injection (sg-HBO1) (Supplementary Figure 1).

HE staining in Figure 1(d) shows that, the liver tissue of the mice showed obvious liver damage in the CCL4-treated group, which was mainly manifested by disordered arrangement of liver cells. At the same time, the connective tissue in the liver was proliferated and aggregated and showed extensive inflammatory cell infiltration. The above-mentioned results confirmed that the mice in the CCL4 group exhibited the obvious liver fibrosis. HE staining showed that the HBO1 knockdown group could significantly reduce liver damage in the mice with liver fibrosis. The degree of degeneration and necrosis of liver cells was reduced, and the infiltration of inflammatory cells was significantly reduced. Masson's trichrome and Sirius Red staining results showed that the collagen deposition in the CCL4 group was significantly increased. Compared with the control group, the collagen deposition increased to 2.53 ± 0.32 times (*p* < 0.01), and the HBO1 knockdown group could reduce collagen deposition compared to the control group (*p* < 0.01) (Figure 1(e)). Additionally, liver weight, body weight, and liver weight/body weight were also detected (please see Supplementary Figure 2).

### 3.2. HBO1 Knockdown Inhibited the Expression of Markers of Liver Fibrosis

**Figure 2:**
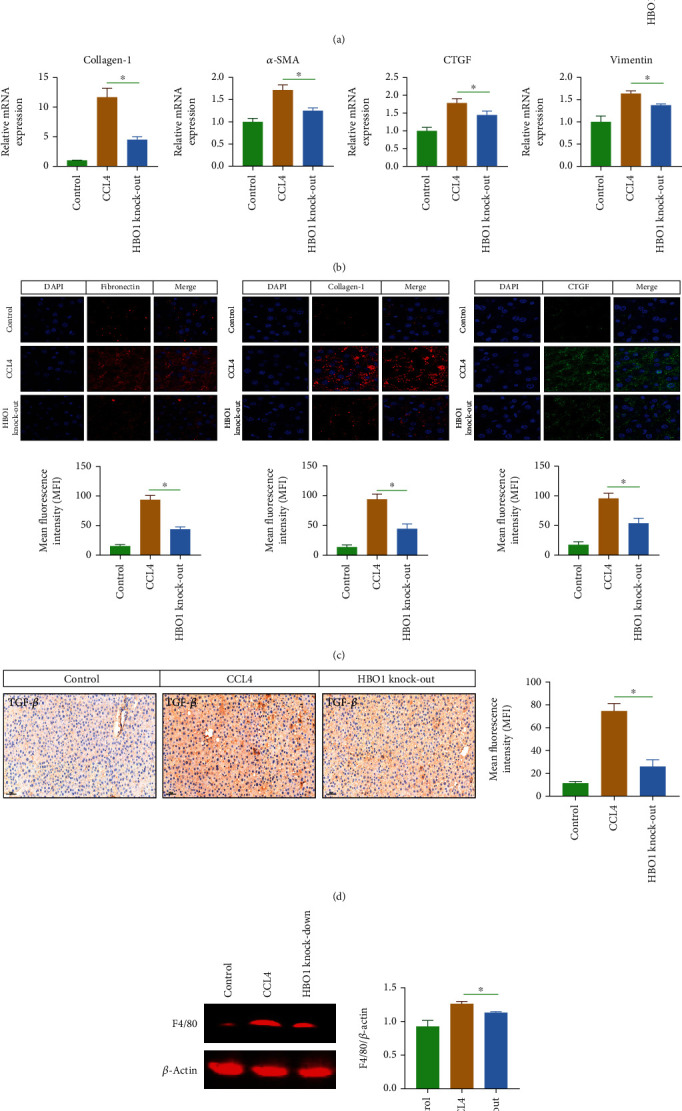
(a) The expression area of *α*-SMA in the HBO1 knockdown group was decreased. The sections were then incubated with 10% normal goat serum and incubated for 1 h for blocking. Primary antibodies were added and incubated overnight at 4°C. Tissue samples were incubated with secondary antibodies. Then, diaminobenzidine (DAB) color development was carried out; the degree of color development was controlled under optical microscope; the samples were dehydrated with gradient ethanol, transparent in xylene, and sealed with neutral gum. Microscopic observation was then performed. (b) Collagen-1, CTGF, *α*-SMA, and vimentin were significantly decreased in HBO1 knockdown group (mRNA level). TRIzol solution was added into the cells or tissues; chloroform was then added at room temperature for 3-5 min. RNA pellets were collected and reverse-transcribed into cDNA. RT-PCR assays were then performed. (c) The expression of collagen-1, CTGF, and fibronectin was downregulated in the HBO1 knockdown group by immunohistochemistry. (d) TGF-*β* expression was significantly decreased in the HBO1 knockdown group. (e) The expression of F4/80 was decreased in HBO1 knockdown group. Data are showed as mean ± standard deviation (SD). Asterisks indicate significant differences (^∗^*p* < 0.05).

Immunohistochemical results showed that the expression of *α*-SMA was significantly increased under the CCL4 induction. Compared with the normal group, the expression area of *α*-SMA in the model group was significantly higher than that in the normal group, and the expression area of *α*-SMA in the HBO1 knockdown group was significantly lower than that of model group (Figure 2(a)). Collagen-1, CTGF, *α*-SMA, and vimentin were significantly decreased in HBO1 knockdown group (mRNA level) (Figure 2(b)). We further analyzed the markers of liver fibrosis, collagen-1, and CTGF are also important marker of liver fibrosis, and their expression levels were significantly increased in CCL4 group. In the HBO1 knockdown group, the expressions of collagen-1 and CTGF were decreased (Figure 2(c)). Immunohistochemical results showed that the expression of fibronectin, CTGF, and collagen-1 in the CCL4 group were obviously decreased (Figure 2(b)). Additionally, we also analyzed the expression of TGF-*β*, and the results showed that TGF-*β* expression was significantly decreased in the HBO1 knockdown group (Figure 2(d)).

In addition, proinflammatory cytokines produced by liver cells can promote the development of liver fibrosis. F4/80 acts as a marker of macrophages, and when F4/80 is highly expressed, it represents an increase in macrophages. The liver macrophages located in the liver sinusoids are important defense cells for regulating immunity, preventing viral and bacterial infections, defending against liver damage, and clearing inflammatory infiltration. When CCL4 induces liver inflammation, macrophages will rush to the site of inflammation to phagocytose cell debris and pathogens. In the current work, the expression of F4/80 was increased in the CCL4 group. But in the HBO1 knockdown group, it can be found that the expression of F4/80 was decreased compared to control group (Figure 2(e)).

### 3.3. Determination of Relevant Biochemical Indicators in the Serum

**Figure 3:**
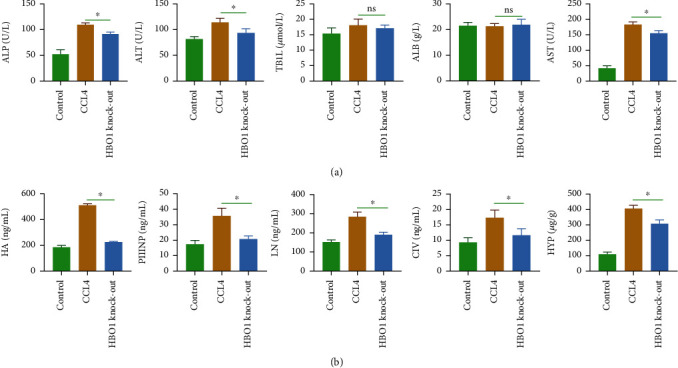
(a) The effect of HBO1 knockdown on the content of ALT/AST. (b) The effect of HBO1 knockdown on the serum levels of HA, PIIINP, LN, CIV, and HYP (liver tissue) in the HBO1 knockdown group. The serum levels of HA, PIIINP, LN, CIV, and HYP were detected by commercialized ELISA kits. Data are showed as mean ± standard deviation (SD). Asterisks indicate significant differences (^∗^*p* < 0.05).

Under the induction of CCL4, the content of ALT/AST in the serum of mice was significantly increased. The serum ALT/AST content of the model group was significantly higher than that of the control group, and the HBO1 knockdown group significantly reduced the ALT/AST serum level. These findings showed that ALT/AST content in HBO1 group was decreased compared with CCL4 (Figure 3(a)). ALT and AST are the indicator of liver function, and its serum level is significantly increased when liver damage is even more severe; these results indicated that HBO1 showed the therapeutic effect on CCL4-induced liver fibrosis. In addition, no significant difference could be observed in serum total bilirubin (TBiL) and albumin (ALB) in all groups.

Next, the changes in serum levels of liver fibrosis markers (HA, PIIINP, LN, and CIV) were determined; we found that the serum levels of HA, PIIINP, LN, and CIV in the CCL4-treated group were significantly increased. But in the HBO1 group, the serum levels of HA, PIIINP, LN, and CIV in the HBO1 knockdown group were remarkably decreased compared to control group (Figure 3(b)).

### 3.4. HBO1 Knockdown Inhibited EMT in Liver Tissue

**Figure 4:**
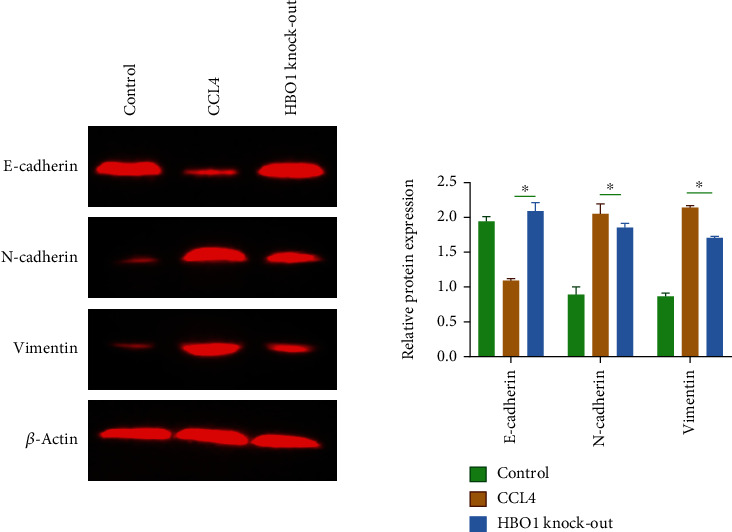
HBO1 knockdown inhibited the occurrence and progression of EMT in CCL4-induced liver fibrosis mice. The protein samples were then subjected to SDS-PAGE. The immunoprotein bands were exposed using Bio-Rad fluorescence system. Data are showed as mean ± standard deviation (SD). Asterisks indicate significant differences (^∗^*p* < 0.05).

EMT (Epithelial-Mesenchymal Transition) plays an important role in the process of liver fibrosis [13, 14]. In the current work, we studied the effect of HBO1 knockdown on the EMT progression in the liver, and we analyzed the expressions of EMT-related markers. The results of western blot showed that the obvious EMT progression occurred in the mouse liver under the CCL4 induction. Compared with the normal group, the expression of N-cadherin and vimentin was increased; the expression of E-cadherin was downregulated. In contrast, in the HBO1 knockdown group, the expressions of N-cadherin and vimentin were significantly downregulated. While E-cadherin was significantly upregulated (*p* < 0.05) (Figure 4). These data collectively suggest that HBO1 knockdown inhibited the occurrence and progression of EMT in CCL4-induced liver fibrosis mice.

### 3.5. HBO1 Knockdown Significantly Reduced the Level of Inflammation in the Liver

**Figure 5:**
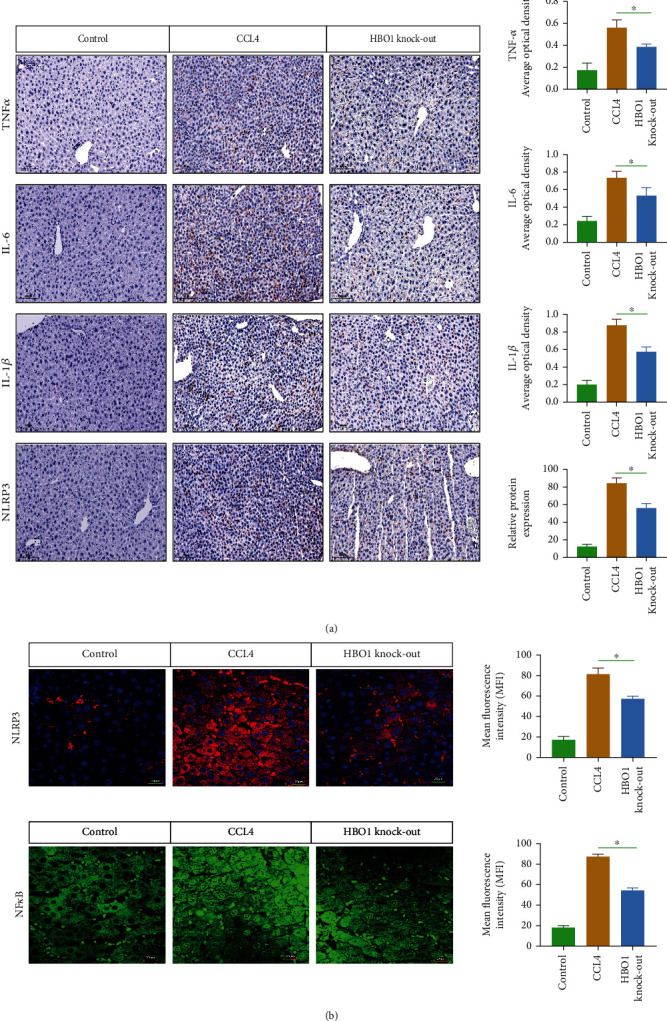
(a) The Knodell score (Knodell histological activity index) showed that the necroinflammationin the HBO1 knockdown group was lower than that of CCL4 group. The sections were them incubated with 10% normal goat serum and incubated for 1 h for blocking. Primary antibodies were added and incubated overnight at 4°C. Tissue samples were incubated with secondary antibodies. Then, diaminobenzidine (DAB) color development was carried out; the degree of color development was controlled under optical microscope; the samples were dehydrated with gradient ethanol, transparent in xylene, and sealed with neutral gum. Microscopic observation was then performed. (b) HBO1 could reduce the expression of NLRP3 and NF-*κ*B. Data are showed as mean ± standard deviation (SD). Asterisks indicate significant differences (^∗^*p* < 0.05).

Tissue damage and inflammatory responses are important triggers of fibrosis [15]. Therefore, we analyzed the effect of HBO1 knockdown on inflammation. It was found that the levels of TNF-*α*, IL-6, and IL-1*β* were significantly increased in the CCL4-treated group, while the levels of inflammation were significantly downregulated in the liver in the HBO1 knockdown group (Figure 5(a)). In addition, we checked the expression levels of NLRP3 and NF-*κ*B, and results showed that HBO1 could significantly reduce the expression of NLRP3 and NF-*κ*B (Figure 5(b)). These results suggest that HBO1 knockdown could significantly reduce the level of inflammation.

### 3.6. HBO1 Knockdown Significantly Reduced the Level of Oxidative Stress in the Liver

**Figure 6:**
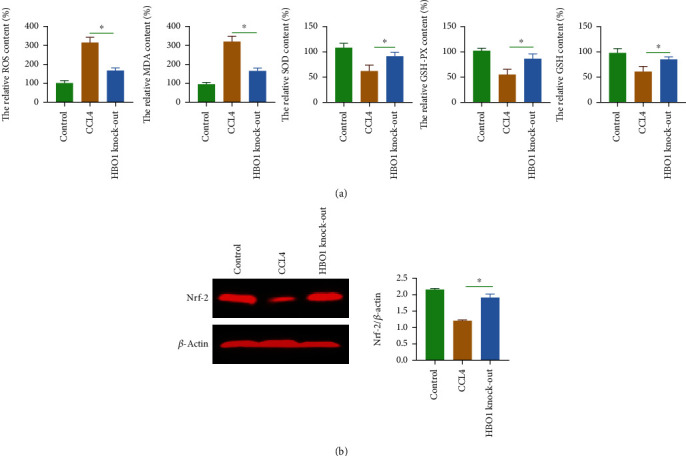
(a) HBO1 knockdown reduced the level of oxidative stress in the liver. Oxidative stress levels were analyzed using a commercially available kit according to the kit's instructions. (b) The effect of HBO1 knockdown on NRF2. The immunoprotein bands were exposed using Bio-Rad fluorescence system. Data are showed as mean ± standard deviation (SD). Asterisks indicate significant differences (^∗^*p* < 0.05).

It has been reported that oxidative stress levels are closely related to liver fibrosis [16]. In the CCL4-induced mouse liver fibrosis group, HBO1 knockdown inhibited the production of ROS. The results of ROS staining showed that the level of ROS was significantly increased under the induction of CCL4. The generation of ROS was significantly higher than that of the normal group (*p* < 0.05). In contrast, SOD activity, GSH-Px, and GSH levels were decreased. Furthermore, MDA levels were also increased in the CCL4-treated group. However, in the HBO1 knockdown group, the levels of ROS and MDA in the liver were decreased, while SOD activity, GSH-Px, and GSH were increased compared with the CCL4 treatment group (*p* < 0.05) (Figure 6(a)). We further analyzed the underlying mechanism by which HBO1 is able to regulate oxidative stress. Western blot results showed that CCL4 significantly reduced the expression of NRF2 (*p* > 0.05). Compared with the model group, the expression of NRF2 (it is an important antioxidant molecule) in the liver was significantly increased in the HBO1 knockdown group, with statistical significance (*p* < 0.05) (Figure 6(b)). These results suggest that HBO1 increases the expression of antioxidant proteins in the CCL4-induced liver fibrosis model.

### 3.7. Analysis of the Underlying Molecular Mechanisms by which HBO1 Regulates Liver Fibrosis *In Vitro*

The liver is composed of a variety of cells. Eighty percent of the liver is liver parenchyma cells. Liver parenchyma cells are differentiated cells that mainly undertake various physiological functions of the liver. The remaining cells, approximately 20%, are hepatic nonparenchymal cells, mainly composed of hepatic stellate cells, sinusoidal endothelial cells, and Kupffer cells [17]. Therefore, these cells are all involved in the process of liver fibrosis. Hepatic stellate cells (HSCs) are very important in the process of liver fibrosis. Under normal conditions, HSCs are in the quiescent state in the liver, and when profibrogenic factors are present, hepatic stellate cells are activated and transformed into myofibroblasts and express *α*-SMA. The activated HSCs proliferate and secrete ECM excessively, which eventually leads to the abnormal accumulation of collagen, and eventually leads to the occurrence of liver fibrosis. We investigated the molecular mechanism of HBO1 antifibrosis from the following aspects:

#### 3.7.1. The Experimental Results *In Vivo* Have Confirmed that HBO1 Knockdown Exhibited the Inhibitory Effect on Liver Fibrosis

**Figure 7:**
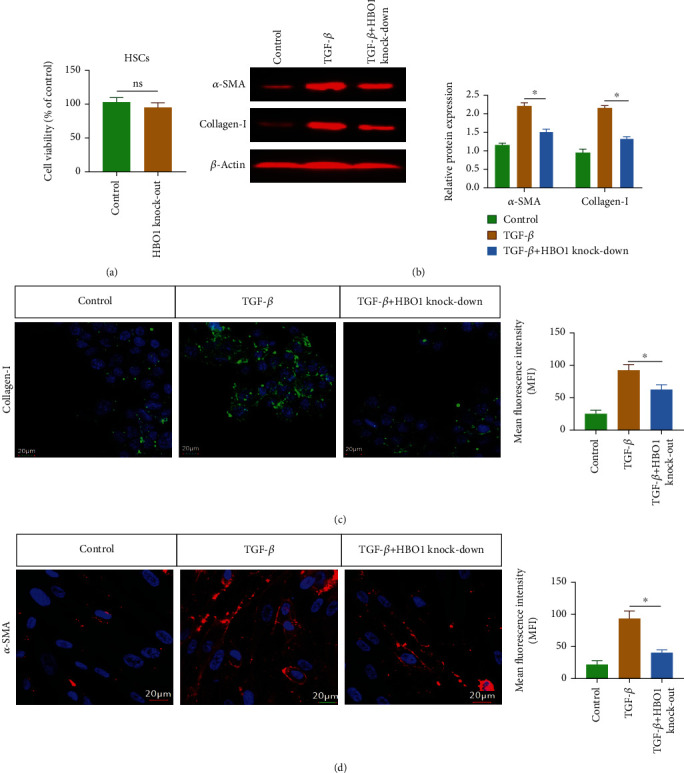
(a) Knockdown of HBO1 did not inhibit the growth of HSCs. (b) HBO1 knockdown could suppress the expression of *α*-SMA and collagen-І. The separated proteins were transferred to PVDF membrane. The immunoprotein bands were exposed using Bio-Rad fluorescence system. (c, d) The expression of *α*-SMA and collagen-І was decreased after HBO1 knockdown. Data are showed as mean ± standard deviation (SD). Asterisks indicate significant differences (  ^∗^*p* < 0.05).

Here, we ask whether HBO1 has a regulatory effect on HSCs *in vitro*. For this, we carried out *in vitro* experiments. MTT was performed to examine the effect of HBO1 knockdown on the cell viability of HSCs. As shown in Figure 7(a), the knockdown of HBO1 (Supplementary Figure 3) did not significantly inhibit the growth of HSCs compared with the normal (blank) control group. The cell viability experiment results indicated that the knockdown of HBO1 did not have obvious toxic effects on HSCs and did not affect the normal growth and survival of HSCs. On this basis, TGF-*β* was used to stimulate HSCs to establish an HSC activation model, and the results showed that the expressions of *α*-SMA and collagen-І were significantly increased in activated HSCs compared with the normal group. HBO1 knockdown could significantly reverse the expression of *α*-SMA and collagen-І (Figure 7(b)). In addition, the immunofluorescence staining was performed to examine the effect of HBO1 knockdown on *α*-SMA and collagen-І in the activated HSCs. Compared with the control group, the TGF-*β* stimulation increased the expression of *α*-SMA and collagen-І, while in the HBO1 knockdown group, the expression of *α*-SMA and collagen-І was significantly decreased (Figures 7(c) and 7(d)); the results of fluorescent staining were consistent with those of western blotting. These results suggest that HBO1 knockdown could significantly inhibit the production of fibrosis marker proteins in the activated HSCs.

#### 3.7.2. HBO1 Knockdown Alleviated CCL4-Induced Cytotoxicity in Hepatocytes and AML12 Cells

**Figure 8:**
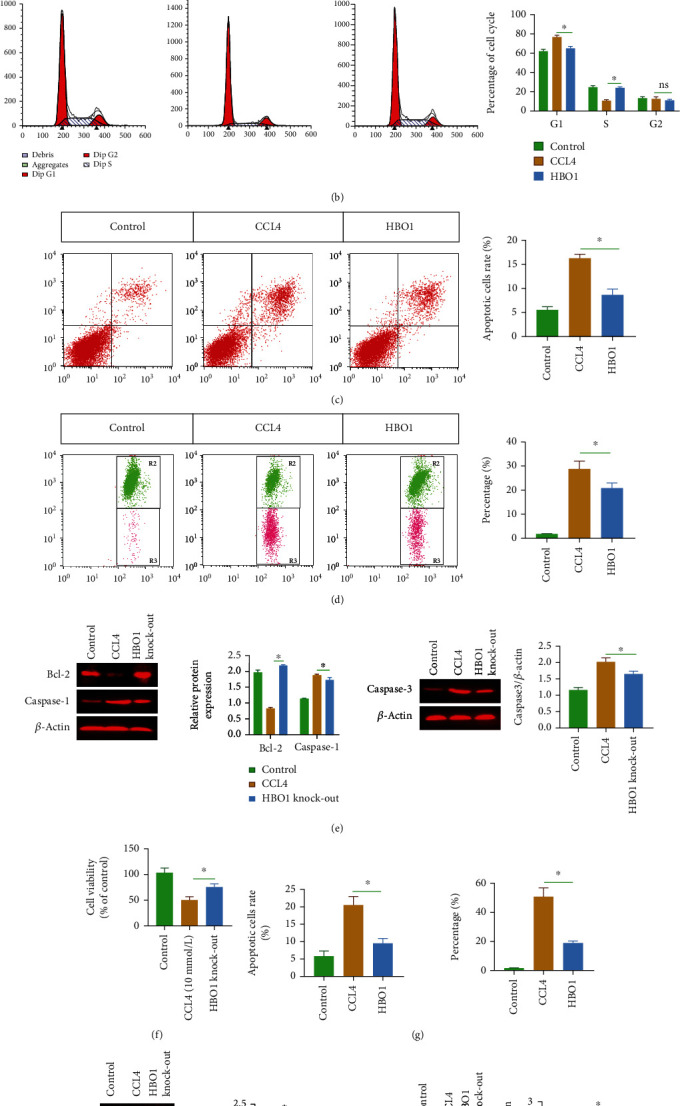
(a) The effect of HBO1 knockdown on CCL4-induced liver cytotoxicity. (b) The proportion of cells in S phase was increased. Cells were collected by centrifugation. The supernatant was then discarded. The cell pellet was homogeneously pipetted, and the cell samples were fixed with fixative solution at 4°C overnight. Cell cycle was detected by flow cytometry. (c) Hepatocyte apoptosis was significantly reduced in the HBO1 knockdown group. (d) The mitochondrial membrane potential was decreased in the HBO1 knockdown group. (e) HBO1 significantly decreased the expression of caspase-1, caspase-3, and Bax and upregulated the expression of Bcl-2. (f) HBO1 knockdown significantly attenuated CCL4-induced cytotoxicity in the AML12 cell line. (g) HBO1 knockdown treatment reduced the rate of cell apoptosis. (h) HBO1 knockdown decreased the expression of Bax, caspase-3, and caspase-1 and increased Bcl-2 expression. Data are showed as mean ± standard deviation (SD). Asterisks indicate significant differences (^∗^*p* < 0.05).

Substances released by apoptotic or pyroptotic hepatocytes are one of the important factors leading to liver fibrosis. Therefore, freshly isolated hepatocytes were used to investigate the effect of HBO1 knockdown on CCL4-induced hepatic cytotoxicity. MTT results showed that when hepatocytes were treated with CCL4 (10 mmol/l), the cell viability of hepatocytes was obviously decreased. However, in the HBO1 knockdown group, the effect of CCL4-induced liver cytotoxicity was significantly reduced (Figure 8(a)). In addition, the cell cycle was significantly altered; the proportion of cells in G0/G1 phase was considerably raised, while the proportion of S phase was significantly decreased in the CCL4 treatment group; while in the HBO1 knockdown group, the proportion of cells in S phase was significantly increased (Figure 8(b)). Further, we detected the cell apoptosis using flow cytometry, and the results showed that compared with the control group, CCL4 treatment significantly increased hepatocyte apoptosis rate; in contrast, hepatocyte apoptosis was significantly reduced in the HBO1 knockdown group, indicating that the HBO1 knockdown group protected against CCL4-induced hepatocyte apoptosis (Figure 8(c)). Furthermore, the results from mitochondrial membrane potential also showed that HBO1 could attenuate CCL4-induced hepatocyte apoptosis (Figure 8(d)). In addition, we also detected the apoptosis-related signaling molecules, and the results showed that CCL4 stimulation significantly increased the expression of caspase-1, caspase-3, and Bax. In contrast, HBO1 significantly decreased the expression of caspase-1, caspase-3, and Bax, and upregulated the expression of Bcl-2 (Figure 8(e)). In addition to a newly isolated hepatocyte cell line model *in vitro*, we also used a mouse hepatocyte cell line (AML12) to further evaluate the effect of HBO1 knockdown (Supplementary Figure 4) on CCL4-induced cytotoxicity. We used the MTT assay to assess the effect of HBO1 on CCL4-induced cytotoxicity in the AML12 cell line. As shown in Figure 8(f), when AML12 was stimulated with CCL4 (10 mmol/l), the cell viability of AML12 was obviously decreased. However, HBO1 knockdown significantly attenuated CCL4-induced cytotoxicity. In addition, cell apoptosis and mitochondrial membrane potential assays also showed that HBO1 knockdown treatment reduced the rate of cell apoptosis (Figure 8(g)). In addition, we also analyzed the expression of Bcl-2 and Bax as well as cleaved-caspase-3 and caspase-1, and the results showed that HBO1 knockdown decreased the expression of Bax, caspase-3, and caspase-1 and increased Bcl-2 expression (Figure 8(h)).

#### 3.7.3. HBO1 Knockdown Alleviated CCL4-Induced Oxidative Stress in Hepatocytes

**Figure 9:**
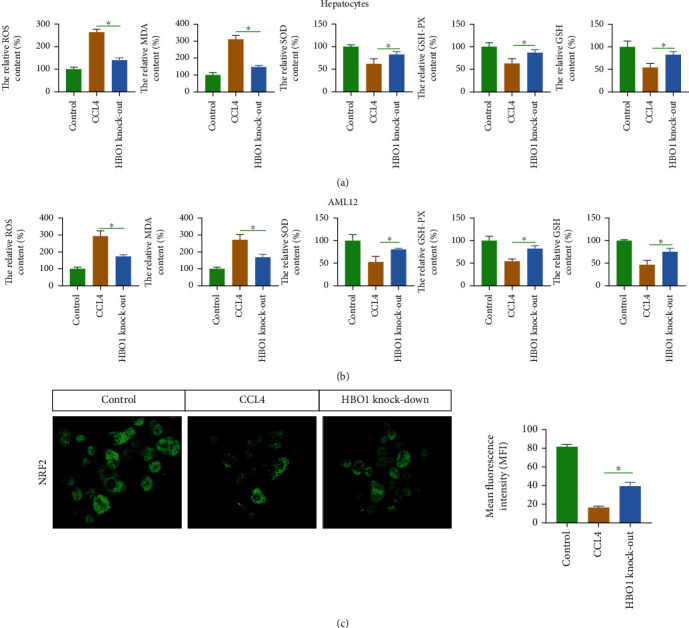
(a) HBO1 knockdown alleviated CCL4-induced oxidative stress in hepatocytes. Oxidative stress levels were analyzed using a commercially available kit according to the kit's instructions. (b) HBO1 knockdown alleviated CCL4-induced oxidative stress in the AML12 cells. (c) HBO1 knockdown upregulated NRF2 expression. The cells were seeded in a 15 mm petri dish. After adherence, cells were cultured for 24 h. The cells were washed three times with PBS and fixed with paraformaldehyde for 15 min at room temperature. 50 *μ*l of 5% goat serum was added to each well of the cell culture plate for 30 min, 50 *μ*l/well of primary antibodies. The next day, the primary antibody was removed. After washing 3 times with PBS, the corresponding fluorescently labeled secondary antibody was added and incubated for 1 h. Finally, nuclei were stained with DAPI. Cell information was collected under an inverted microscope and confocal microscope. Data are showed as mean ± standard deviation (SD). Asterisks indicate significant differences (^∗^*p* < 0.05).

Previous studies have reported that CCL4 induces the accumulation of free radicals in hepatocytes, leading to oxidative stress injury [18]. Thus, to further study the biological basis of the protective effect of HBO1 on hepatocytes, we analyzed the effect of HBO1 on CCL4-induced hepatic oxidative damage.  Figure 9(a) indicates that the ROS level was increased about 2-fold compared with the control group. In addition, SOD activity, GSH-Px, and GSH levels were decreased, and MDA levels were increased in CCL4-treated hepatocytes. However, HBO1 knockdown reduced intracellular ROS and MDA levels. Furthermore, the HBO1 knockdown group prevented the CCL4-induced reduction of SOD, GSH-Px, and GSH (*p* < 0.05).

Next, we also performed the same experiments on mouse liver cell lines (AML12) and found that HBO1 knockdown could affect oxidative damage in the AML12 cell line. SOD activity, GSH-Px, and GSH levels were decreased, and MDA levels were increased in CCL4-treated AML12. In contrast, the HBO1 knockdown group prevented the CCL4-induced reduction of SOD, GSH-Px. and GSH levels. In addition, the HBO1 knockdown group decreased the ROS and MDA levels (*p* < 0.05) (Figure 9(b)).

To analyze the molecular mechanism by which HBO1 knockdown alleviates oxidative stress, it is well known that NRF2 is involved in the process of oxidative stress. It has been reported that NRF2 could regulates the expression of antioxidant genes and chemokine-related genes, which in turn inhibits cellular oxidative damage [19]. In the current work, we found that after the primary hepatocytes were treated with CCL4, the NRF2 expression was decreased. In HBO1 knockdown group, NRF2 significantly upregulated (Figure 9(c)). These experiments suggest that HBO1 may play an antioxidative stress role by upregulating NRF2.

#### 3.7.4. Knockdown of HBO1 Alleviated LPS-Induced Inflammatory Response in Liver Cells

**Figure 10:**
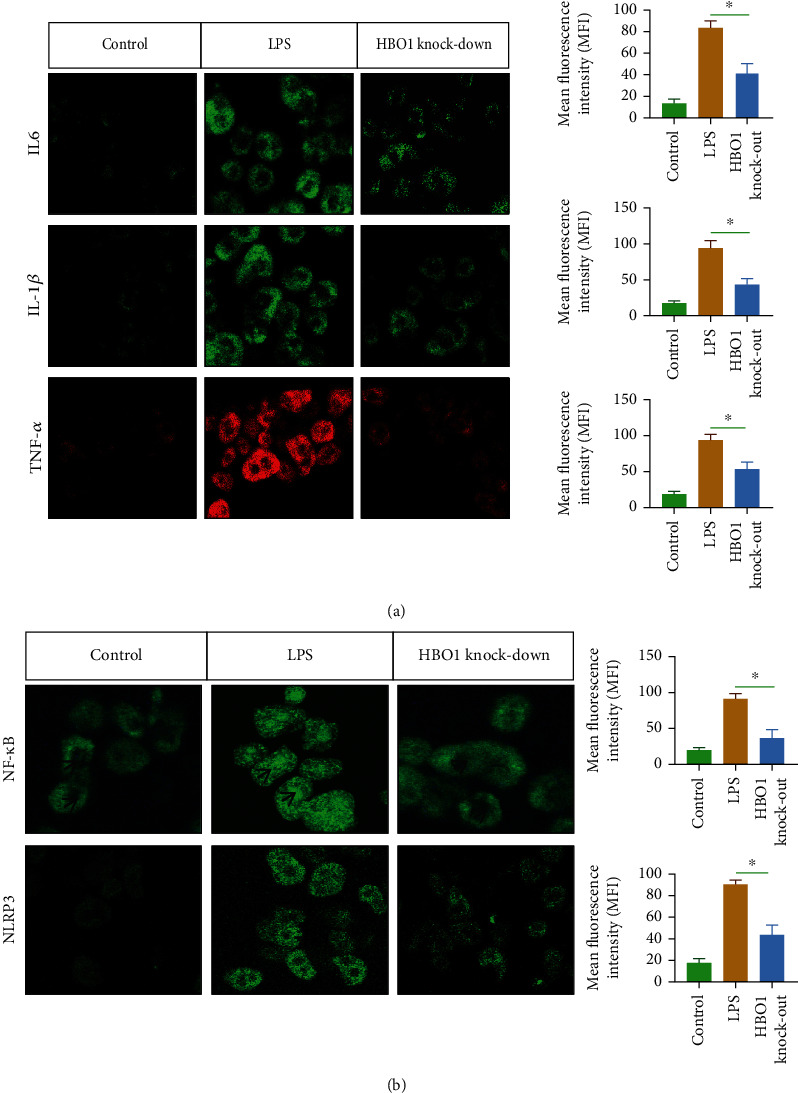
(a) Inflammation level was significantly downregulated in HBO1 knockdown group. The cells were seeded in a 15 mm petri dish. After adherence, cells were cultured for 24 h. The cells were washed three times with PBS and fixed with paraformaldehyde for 15 min at room temperature. 50 *μ*l of 5% goat serum was added to each well of the cell culture plate for 30 min, 50 *μ*l/well of primary antibodies. The next day, the primary antibody was removed. After washing 3 times with PBS, the corresponding fluorescently labeled secondary antibody was added and incubated for 1 h. Finally, nuclei were stained with DAPI. Cell information was collected under an inverted microscope and confocal microscope. (b) The expression of NF-*κ*B signaling and NLRP3 were reduced compared to LPS treatment group. Data are showed as mean ± standard deviation (SD). Asterisks indicate significant differences (^∗^*p* < 0.05).

We further investigated the effect of HBO1 on LPS-induced inflammatory cell model. In the freshly isolated primary hepatocyte model from sg-HBO1 mice. The hepatocytes were stimulated with LPS (1000 ng/ml), and results showed that LPS stimulation activated a series of inflammatory molecules expression, including IL-6, IL-1*β*, and TNF-*α*. In the HBO1 knockdown group, and inflammation level was substantially reduced compared with LPS treatment group (Figure 10(a)). In addition, the results indicated that NF-*κ*B signaling was downregulated. Additionally, NLRP3 expression was also reduced compared to LPS treatment group (Figure 10(b)).

### 3.8. HBO1 Knockdown Alleviated Liver Aging and Aging-Related Liver Fibrosis

**Figure 11:**
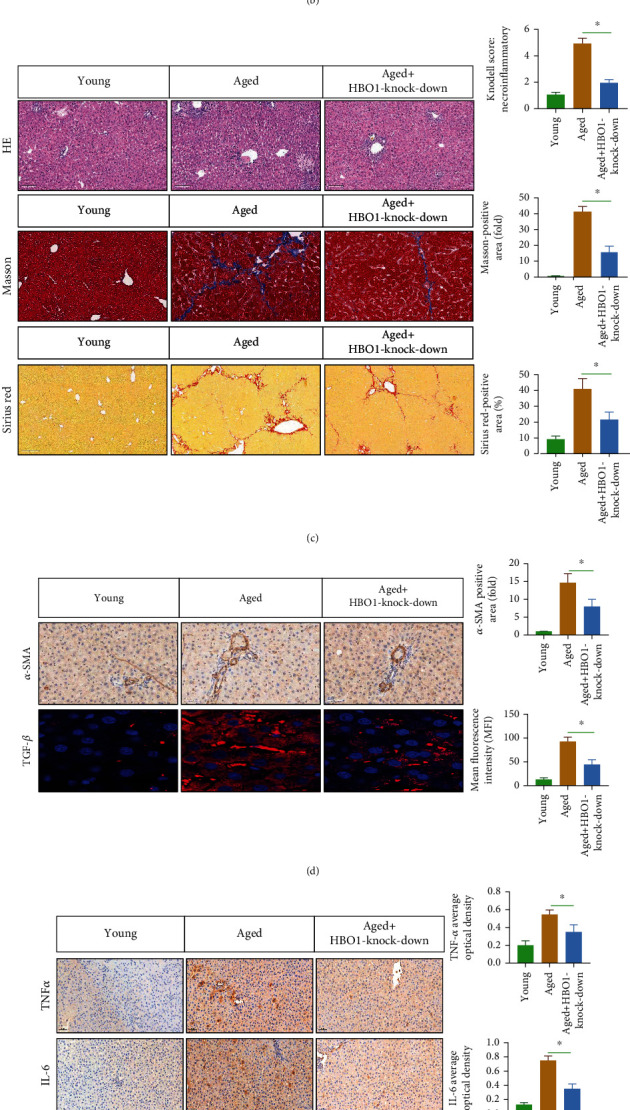
(a) HBO1 expression was significantly decreased in HBO1 knockdown group. (b) HBO1 knockdown alleviated the liver aging. (c) The level of liver fibrosis was alleviated in HBO1 knockdown group. (d) TGF-*β* and *α*-SMA expressions were reduced. (e) HBO1 knockdown reduced the expression of proinflammatory molecules (TNF-*α* and IL6). (f) Rotarod and running wheel results show greater exercise capacity in the HBO1 knockout group that that of control group. (g) HBO1 knockdown improved on memory and learning ability of aged mice. Data are showed as mean ± standard deviation (SD). Asterisks indicate significant differences (^∗^*p* < 0.05).

Studies have indicated that aging is closely related to liver fibrosis [20]. We used 18-month-old mice as in vivo model, the aged mice were injected with lentivirus (sg-HBO1) via tail vein and harvested liver tissue for observation 6-8 weeks later. We first found that HBO1 was highly expressed in the aged mice. However, in the sg-HBO1 lentivirus group, HBO1 expression was significantly decreased (Figure 11(a)). HBO1 knockdown alleviated the liver aging (Figure 11(b)). Additionally, we analyzed and compared the liver fibrosis between aged mice and sg-HBO1 group. Masson and Sirius Red staining indicated that the level of liver fibrosis was alleviated in HBO1 knockdown group (Figure 11(c)). Furthermore, TGF-*β* and *α*-SMA expressions significantly reduced (Figure 11(d)). In addition, HBO1 knockdown also significantly reduced the expression of proinflammatory molecules (TNF-*α* and IL-6) (Figure 11(e)). We further analyzed the effect of HBO1 on animal behavior from the perspective of animal behavior. Rotarod and running wheel results show greater exercise capacity in the HBO1 knockout group that that of control group (Figure 11(f)). Y-maze and open field test experiments were conducted to study the effects of HBO1 knockdown on memory and learning in mice, and the results showed that HBO1 knockdown improved on memory and learning ability of aged mice (Figure 11(g)).

**Figure 12:**
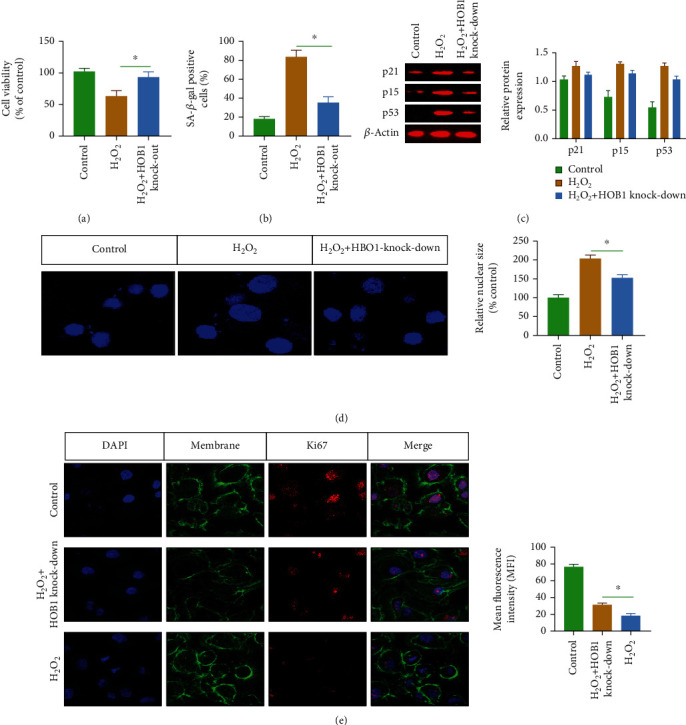
(a) H_2_O_2_ treatment reduced the cell viability. (b) The number of SA-*β*-gal-positive cells was significantly increased. (c) The expressions p16, p53, and p21 were decreased in HBO1 knockdown group. The separated proteins were transferred to PVDF membrane. After blocking, diluted primary antibodies were added to the membranes and incubated overnight at 4°C. After primary antibody incubation, BSA-diluted secondary antibody was added and incubated for 2 h at 37°C. The immunoprotein bands were exposed using Bio-Rad fluorescence system. (d) The size of the nucleus was analyzed. (e) Ki67 expression increased in the HBO1 knockdown group. Data are showed as mean ± standard deviation (SD). Asterisks indicate significant differences (^∗^*p* < 0.05).

To explore the antiaging effect of HBO1 knockdown, an aging model of liver cell (AML12) was established by using H_2_O_2_. For this, the AML12 cells were treated with different concentrations of H_2_O_2_ (0-100 *μ*mol) to induce AML12 cellular senescence. The MTT assay was conducted to test the effect of various concentrations of H_2_O_2_ on cell viability, and the results indicated that H_2_O_2_ treatment reduced the cell viability (Figure 12(a)). Additionally, the number of SA-*β*-gal-positive cells increased (Figure 12(b)). Combining MTT assays and SA-*β*-gal staining results, 50 *μ*m H_2_O_2_ was selected for the following experiments.

HBO1 knockdown (please see Supplementary Figure 5) reduced the number of SA-*β*-gal-positive AML12 cell. MTT assays showed that HBO1 knockdown increased the cell viability of AML12. The expression p16, p53, and p21 were decreased compared to H_2_O_2_ group (Figure 12(c)). The size of the nucleus was also analyzed (Figure 12(d)). Ki67 expression was also increased in the HBO1 knockdown group (Figure 12(e)).

## 4. Discussion

Liver fibrosis is a serious threat to human health [21]. There are many factors that cause liver fibrosis. Liver fibrosis is caused by chronic liver injury and accumulation of ECM, which is also characteristic of most chronic liver diseases [22]. Liver fibrosis is also a dynamic process, with the participation of different types of cell groups to maintain and coordinate the occurrence of liver fibrosis, and a series of biologically active factors (such as growth factors and cytokines) also involves in the process of liver fibrosis. Aging is also one of the important pathogenic factors of liver fibrosis. There are currently three mainstream diagnostic methods for liver fibrosis, namely, liver-related pathological diagnosis, liver-related imaging diagnosis, and liver-related serological diagnosis. But so far, in the face of liver fibrosis, humans have not been able to find a better way to treat liver fibrosis. Therefore, finding effective potential treatments for liver fibrosis is an urgent scientific problem to be solved. In the current study, we found that HBO1 could serve as a target to treat liver fibrosis and liver aging.

HBO1 has been reported to have many biological activities [23]. In this work, we mainly evaluated the effect of targeting HBO1 in the treatment of liver fibrosis. For this, we first established a CCL4-induced liver fibrosis model and found that HBO1 knockdown exhibited a better therapeutic effect on CCL4-induced liver fibrosis. *In vivo* experiments, the results of Masson and Sirius Red staining showed that knockdown of HBO1 could significantly inhibit the deposition of collagen. Furthermore, the expression of *α*-SMA and collagen-1 was significantly decreased. In addition, serum AST/ALT levels were also decreased *in vivo*. These findings show that HBO1 can significantly inhibit and relieve the live fibrosis.

The liver is composed of a variety of cells, 80% of which are liver parenchyma cells, which are differentiated cells that mainly undertake various physiological functions of the liver. The remaining about 20% are liver nonparenchymal cells, mainly including hepatic stellate cells, hepatic sinusoidal endothelial cells, and Kupffer cells. Therefore, these cells are all involved in the process of liver fibrosis. Stellate cells (HSCs) are considered the most important cell in the process of liver fibrosis. Under normal conditions, HSCs are in quiescent state in the liver. However, when profibrogenic factors are present, activated HSCs transform into myofibroblasts and express *α*-SMA and secrete ECM excessively, which induces the abnormal accumulation of collagen, and eventually leads to the occurrence of liver fibrosis. In the process of liver fibrosis, HSC activation is a core event. It has been reported that TGF-*β*/Smad signaling pathway, EMT process, and chronic inflammation are all involved in the occurrence and development of liver fibrosis [24]. To evaluate the effect of HBO1 knockdown on HSC activation, a series of *in vitro* and *in vivo* experiments were performed. It was found that HBO1 knockdown decreased the expression of *α*-SMA. Previous studies have shown that KAT7 promotes histone acetylation of TGF-*β* promoter to enhance TGF-*β* expression. In addition, the expressions of collagen-1 and CTGF were also significantly reduced. HSC can be a target for the treatment of liver fibrosis. For example, the blockade of YAP could alleviate hepatic fibrosis via inhibiting HSC activation [24]. Additionally, aspirin could alleviate hepatic fibrosis via inhibiting HSC activation though NF-*κ*B signaling pathway [25]. Metformin also could inhibit activation of hepatic stellate cells [26].

Inflammation plays an important role in the pathophysiological process of liver fibrosis. Notably, there is growing evidence that inflammation is thought to be an important factor for liver disease. Inflammation can activate innate immune cells to produce proinflammatory cytokines (such as IL-1*α*, IL-1*β*, and TNF-*α*). Continuous stimulation of proinflammatory factors can induce the activation of HSCs, leading to the occurrence of chronic inflammatory liver fibrosis [25]. Based on the effect of inflammation on liver fibrosis, inhibiting the production of inflammation may be a potential method to alleviate the development of liver fibrosis. In the current study, we found that downregulation of HBO1 can significantly downregulate the expression of proinflammatory factors (such as IL-6, TNF-*α*, and IL-1*β*). In addition, we analyzed the levels of NLRP3 and NF-*κ*B and found that HBO1 knockdown could significantly reduce the expression of NLRP3 and NF-*κ*B, suggesting that HBO1 knockdown can significantly reduce the level of inflammation. Previous studies have shown that KAT7 promotes histone acetylation of IL-6 promoter and enhances IL-6 expression [27].

Epithelial-mesenchymal transformation (EMT) is a process that occurs in various tissues or organs involved in tissue development and wound healing; epithelial cells lose their cellular identity and exhibit the properties of mesenchymal-like cells. When EMT occurs, the expression of cell adhesion molecules (such as E-cadherin) is reduced, and E-cadherin is replaced by proteins that provide greater connection flexibility (such as N-cadherin). The current study showed that HBO1 knockdown was able to inhibit the EMT transformation of HSCs. Recent studies have shown that HBO1 can promote the progress of EMT by regulating Wnt/*β*-catenin signaling pathway [28].

Oxidative stress is closely related with liver fibrosis. Here, we found that oxidative stress was substantially decreased in the HBO1 group, and the level of reactive oxygen species (ROS) was decreased. Further studies found that the expression of antioxidant signaling molecule (NRF2) was significantly upregulated. Recent studies have reported that HBO1 is closely related to oxidative stress, and knockdown of HBO1 can alleviate oxidative stress [29]. In the current study, we found that HBO1 may regulate oxidative stress by regulating NRF2.

Aging is also one of the important factors of liver fibrosis. In the current study, we found that HBO1 knockdown could alleviate liver aging and fibrosis *in vivo* and *in vitro*.

In conclusion, we found that HBO1 can act as a target to treat liver fibrosis. This work indicates that HBO1 is a new target for liver fibrosis treatment.

## Data Availability

All data can be obtained from the corresponding author based on reasonable request.

## Supplementary Materials

Supplementary Materials Supplementary Figure 1: HBO1 expression was evaluated after lentivirus injection. Supplementary Figure 2: detection of liver weight, body weight, and liver weight/body weight. Supplementary Figure 3: knockdown of HBO1 in HSCs. Supplementary Figure 4: knockdown of HBO1 in AML12 cells. Supplementary Figure 5: knockdown of HBO1 in AML12 cells. Supplementary Table 1: primer sequence.

## References

[1] Friedman S. L. (2003). Liver fibrosis - from bench to bedside. *Journal of Hepatology*.

[2] Iredale J., Campana L. (2017). Regression of Liver Fibrosis. *Seminars in Liver Disease*.

[3] Berumen J., Baglieri J., Kisseleva T., Mekeel K. (2021). Liver fibrosis: pathophysiology and clinical implications. *WIREs Mechanisms of Disease*.

[4] Schuppan D., Kim Y. (2013). Evolving therapies for liver fibrosis. *Journal of Clinical Investigation*.

[5] Budi E. H., Schaub J. R., Decaris M., Turner S., Derynck R. (2021). TGF-*β* as a driver of fibrosis: physiological roles and therapeutic opportunities. *The Journal of Pathology.*.

[6] Chen S. R., Chen X. P., Lu J. J., Wang Y., Wang Y. T. (2015). Potent natural products and herbal medicines for treating liver fibrosis. *Chinese Medicine*.

[7] Feng Y., Cheung K. F., Wang N., Liu P., Nagamatsu T., Tong Y. (2009). Chinese medicines as a resource for liver fibrosis treatment. *Chinese Medicine*.

[8] Zhao Z., Lin C. Y., Cheng K. (2019). siRNA- and miRNA-based therapeutics for liver fibrosis. *Translational Research*.

[9] Kueh A. J., Eccles S., Tang L. (2020). HBO1 (KAT7) does not have anessential role in cell proliferation, DNA replication, or histone 4 acetylation in human cells. *Molecular and Cellular Biology*.

[10] Liang Y., Su Y., Xu C. (2020). Protein kinase D1 phosphorylation of KAT7 enhances its protein stability and promotes replication licensing and cell proliferation. *Cell death discovery*.

[11] Xiao Y., Li W., Yang H. (2021). HBO1 is a versatile histone acyltransferase critical for promoter histone acylations. *Nucleic Acids Research*.

[12] Jie M., Wu Y., Gao M. (2020). CircMRPS35 suppresses gastric cancer progression via recruiting KAT7 to govern histone modification. *Molecular cancer*.

[13] Chilvery S., Bansod S., Saifi M. A., Godugu C. (2020). Piperlongumine attenuates bile duct ligation-induced liver fibrosis in mice via inhibition of TGF-*β*1/Smad and EMT pathways. *International Immunopharmacology*.

[14] Yao X., Wang J., Zhu J., Rong X. (2020). The anti-fibrotic effect of human fetal skin-derived stem cell secretome on the liver fibrosis. *Stem Cell Research & Therapy*.

[15] Wan J., Weiss E., Mkaddem S. B. (2020). LC3-associated phagocytosis protects against inflammation and liver fibrosis via immunoreceptor inhibitory signaling. *Science Translational Medicine*.

[16] Ramos-Tovar E., Muriel P. (2020). Molecular mechanisms that link oxidative stress, inflammation, and fibrosis in the liver. *Antioxidants*.

[17] Ozawa S., Miura T., Terashima J., Habano W., Ishida S. (2021). Recent progress in prediction pystems for drug-induced liver injury using in vitro cell culture. *Drug Metabolism Letters*.

[18] Wang T., Zhou X., Kuang G. (2021). Paeoniflorin modulates oxidative stress, inflammation and hepatic stellate cells activation to alleviate CCl4-induced hepatic fibrosis by upregulation of heme oxygenase-1 in mice. *Journal of Pharmacy and Pharmacology*.

[19] El-Emam S. Z., Soubh A. A., Al-Mokaddem A. K., Abo El-Ella D. M. (2020). Geraniol activates Nrf-2/HO-1 signaling pathway mediating protection against oxidative stress-induced apoptosis in hepatic ischemia-reperfusion injury. *Naunyn-Schmiedeberg’s Archives of Pharmacology*.

[20] Allaire M., Gilgenkrantz H. (2020). The aged liver: beyond cellular senescence. *Clinics and research in hepatologyand gastroenterology*.

[21] Roehlen N., Crouchet E., Baumert T. F. (2020). Liver fibrosis: mechanistic concepts and therapeutic perspectives. *Cell*.

[22] Kisseleva T., Brenner D. (2021). Molecular and cellular mechanisms of liver fibrosis and its regression. *Nature Reviews Gastroenterology & Hepatology*.

[23] Han J., Lachance C., Ricketts M. D. (2018). The scaffolding protein JADE1 physically links the acetyltransferase subunit HBO1 with its histone H3-H4 substrate. *Journal of Biological Chemistry*.

[24] Yu H. X., Yao Y., Bu F. T. (2019). Blockade of YAP alleviates hepatic fibrosis through accelerating apoptosis and reversion of activated hepatic stellate cells. *Molecular Immunology*.

[25] Liu Y., Nong L., Jia Y. (2020). Aspirin alleviates hepatic fibrosis by suppressing hepatic stellate cells activation via the TLR4/NF-*κ*B pathway. *Aging (Albany NY)*.

[26] Nguyen G., Park S. Y., Le C. T., Park W. S., Choi D. H., Cho E. H. (2018). Metformin ameliorates activation of hepatic stellate cells and hepatic fibrosis by succinate and GPR91 inhibition. *Biochemical and Biophysical Research Communications*.

[27] Gao S., Qi X., Li J., Sang L. (2017). Upregulated KAT7 in synovial fibroblasts promotes Th17 cell differentiation and infiltration in rheumatoid arthritis. *Biochemical and Biophysical Research Communications*.

[28] Bai Z., Xia X., Lu J. (2020). MicroRNA-639 is down-regulated in hepatocellular carcinoma tumor tissue and inhibits proliferation and migration of human hepatocellular carcinoma cells through the KAT7/Wnt/*β*-catenin pathway. *Medical science monitor: international medical journal of experimental and clinical research*.

[29] Wang B., Li J., Liu L., Song G. (2022). Insulin sensitivity in the aged heart is improved by down-regulation of KAT7 in vivo and in vitro. *Cell Cycle*.

