# Supplementary material for: HBO1 as an Important Target for the Treatment of CCL4-Induced Liver Fibrosis and Aged-Related Liver Aging and Fibrosis
Source: Oxid Med Cell Longev. 2022 Dec 6;2022:1881519. doi: 10.1155/2022/1881519 (PMC9747301; doi:10.1155/2022/1881519)
Supplement: Supplementary Materials — Supplementary Figure 1: HBO1 expression was evaluated after lentivirus injection. Supplementary Figure 2: detection of liver weight, body weight, and liver weight/body weight. Supplementary Figure 3: knockdown of HBO1 in HSCs. Supplementary Figure 4: knockdown of HBO1 in AML12 cells. Supplementary Figure 5: knockdown of HBO1 in AML12 cells. Supplementary Table 1: primer sequence. [file 1881519.f1.docx]

NRF2: Forward, CGAGATATACGCAGGAGAGGTAAGA; Reverse, GCTCGACAATGTTCTCCAGCTT [1];

1. El-Ghaiesh, S. H., Bahr, H. I., Ibrahiem, A. T., Ghorab, D., Alomar, S. Y., Farag, N. E., & Zaitone, S. A. (2020). Metformin protects from rotenone–induced nigrostriatal neuronal death in adult mice by activating AMPK-FOXO3 signaling and mitigation of angiogenesis. Frontiers in molecular neuroscience, 13, 84.
